# Synthesis, Structure, Biological Activity, and Luminescence Properties of a “Butterfly”-Type Silver Cluster with 3-Benzyl-4-phenyl-1,2,4-triazol-5-thiol

**DOI:** 10.3390/molecules29010105

**Published:** 2023-12-23

**Authors:** Dmitriy S. Yambulatov, Irina A. Lutsenko, Dmitry E. Baravikov, Fedor M. Dolgushin, Tatiana V. Astaf’eva, Olga B. Bekker, Lusik G. Nersisyan, Melanya A. Samvelyan, Tariel V. Ghochikyan, Mikhail A. Kiskin, Igor L. Eremenko, Vladimir K. Ivanov

**Affiliations:** 1 Kurnakov Institute of General and Inorganic Chemistry, Russian Academy of Sciences, 31 Leninsky Prosp., 119991 Moscow, Russia; irinalu05@rambler.ru (I.A.L.); dbbaravikov@gmail.com (D.E.B.); fmdolgushin@gmail.com (F.M.D.); tata1525@mail.ru (T.V.A.); m_kiskin@mail.ru (M.A.K.); ileremenko@yandex.ru (I.L.E.); van@igic.ras.ru (V.K.I.); 2 Vavilov Institute of General Genetics, Russian Academy of Sciences, Gubkina, 3, 119333 Moscow, Russia; obekker@yandex.ru; 3 Faculty of Chemistry, Yerevan State University, 1 A Manoukyan Str., Yerevan 0025, Armenia; nersisyanlusik@mail.ru (L.G.N.); msamvelyan@ysu.am (M.A.S.); tghochikyan@gmail.com (T.V.G.)

**Keywords:** 1,2,4-triazole, silver, molecular structure, luminescence, single crystal, metallodrugs, biological activity

## Abstract

A new silver(I) cluster [Ag_8_L_4_(Py)(Pype)]·4Py·11H_2_O (**I**) with 3-benzyl-4-phenyl-1,2,4-triazol-5-thiol (L) was synthesized via the direct reaction of AgNO_3_ and L in MeOH, followed by recrystallization from a pyridine–piperidine mixture. The compound **I** was isolated in a monocrystal form and its crystal structure was determined via single crystal X-ray diffraction. The complex forms a “butterfly” cluster with triazol-5-thioles. The purity of the silver complex and its stability in the solution was confirmed via NMR analysis. Excitation and emission of the free ligand and its silver complex were studied at room temperature for solid samples. The in vitro biological activity of the free ligand and its complex was studied in relation to the non-pathogenic *Mycolicibacterium smegmatis* strain. Complexation of the free ligand with silver increases the biological activity of the former by almost twenty times. For the newly obtained silver cluster, a bactericidal effect was established.

## 1. Introduction

The current approach of advanced bioinorganic chemistry consists of using simple well-known biologically active molecules to design effective metallodrugs. Such ligands include, for example, benzimidazole derivatives [1,2]; purine bases [3,4], particularly caffeine [5,6,7,8,9]; the well-known commercially available ligand 1,10-phenanthroline [10]; anions of biologically active carboxylic acids (benzoic [11,12], 2-furoic [13,14], etc.); dithiocarbamates [15,16]; and redox-active ligands [17,18,19,20,21]. In most cases, after coordination of a biologically active molecule to a metal ion, the bioactivity of the resulting complex increases significantly [22,23,24,25,26], simultaneously reducing the toxicity and, therefore, the lethal dose of an organic ligand, which make it possible to use this substance in therapy.

A well-studied family of biologically active ligands that can be used to create new metallodrugs includes the derivatives of 1,2,4-triazoles, which have been known for more than 100 years and the methods of their synthesis and chemical properties have been studied extensively [27]. Their biological activity has been proven via many years of clinical use as drugs registered in the European Pharmacopoeia, for example, Alprazolam (sedative), Anastrozole (treatment for breast cancer), Brotizolam (sedative), Fluconazole (antifungal), Itraconazole (antifungal), Letrozole (treatment for breast cancer), Ribavirin (antiviral), Terconazole (antifungal), and Trapidi (antiaggregant) [28]. Despite more than a century-long history of studying triazoles, they still attract the attention of researchers [29]. When designing novel bioactive metal complexes, it is very important to know the mechanism of pharmacokinetics; in the case of 1,2,4-triazole derivatives, these data are already available [30,31,32], which can facilitate and reduce the cost of further testing of the corresponding metallodrugs.

To date, reliable data have been obtained on the antifungal, antioxidant, anticonvulsant, anti-inflammatory activity of metal complexes with 1,2,4-triazoles and their potential use for the treatment of tuberculosis, diabetes, and different types of cancer [33,34]. It was shown that triazole-derived Schiff base ligands and their cobalt(II), nickel(II), copper(II), and zinc(II) complexes reveal antibacterial and antifungal activity, of which the study proved that the metal complexes showed better activity than the ligands after coordination [35,36]. It was also shown that the addition of other donor atoms (oxygen, nitrogen, and sulfur) to triazole derivatives leads to the formation of biologically active complexes. For example, vanadyl(IV) complexes with mono- and di-substituted ONS donor triazoles reveal antibacterial, antifungal, and cytotoxic activity [37,38].

According to the previous studies, silver cation is a promising complex-forming ion in the design of metallodrugs [39,40,41,42]. Silver-containing compounds are currently used for the therapy of skin diseases and burns: silver sulfadiazine [39], silver proteinate (Protargol) [43], and silver nitrate [44]. Since silver has long been used in the treatment of various diseases, its pharmacokinetics, like 1,2,4-triazole derivatives, are also well studied [45,46]. Having analyzed the Cambridge structural database, we found that only a few dozen structurally characterized silver complexes with 1,2,4-triazole derivatives are known, and to the best of our knowledge, the biological activity of these compounds have not been studied.

Bioactive luminescent metallodrugs are of great interest in the field of bioinorganic chemistry due to the potential applications of these compounds for the imaging of living cells [47]. This approach can be used to track the distribution of luminescent substances within a cell and study their uptake by cells [48]. Undoubtedly, the leaders in bioimaging among compounds used in modern medicine and biology are complexes based on lanthanides [49,50]. However, currently more and more research is devoted to promising luminescent probes in living systems based on Ag(I) (with simultaneous antibacterial properties). It was found that luminescent properties of Au(I) and Ag(I) complexes with N-heterocyclic carbenes can be used to investigate biodistribution [51].

The previously studied silver complexes with 1,2,4-triazole derivatives exhibit luminescent properties [52,53,54], which are very important in the design of promising metal-containing drugs [55,56,57,58]. It was also previously shown that silver has a high affinity for the sulfur atom, forming strong linear compounds or layers [59,60,61].

**Scheme 1:**
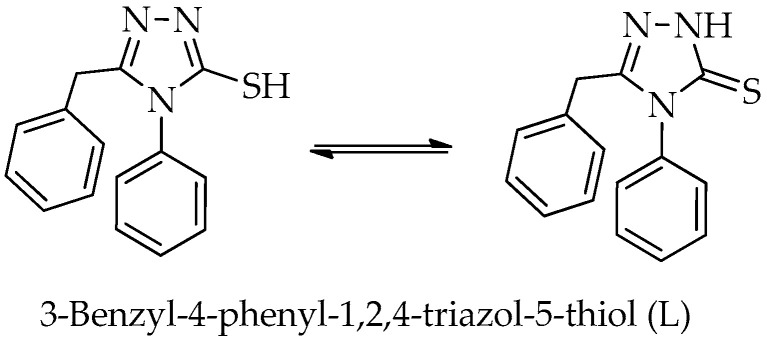
Keto–enol tautomerization of L.

Thus, the goal of this study was to develop a method for the synthesis of a new silver complex with a 1,2,4-triazole sulfur-containing derivative—3-benzyl-4-phenyl-1,2,4-triazol-5-thiol (L, Scheme 1)—and determine its molecular structure, confirm the purity, and analyze the luminescent properties of the free ligand and the complex. Also, the purpose of this study was to find out whether the free ligand and its silver complex exhibit biological activity against *M. Smegmatis* (a model non-virulent strain of *M. tubercolisis*) and how the coordination of the ligand affects these properties. This current work is a part of our ongoing research to find new anti-tuberculosis metallodrugs [22,24,25,26,62].

## 2. Results and Discussion

### 2.1. Synthesis and Characterization

**Scheme 2:**
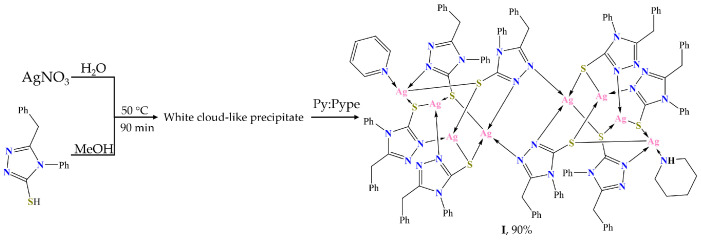
Synthesis route of compound **I**, solvate molecules of water and pyridine have been omitted for clarity.

The reaction of L and AgNO_3_ in a methanol solution with further crystallization from the mixture of pyridine (Py) and piperidine (Pype) (Scheme 2) led to the formation of the product [Ag_8_L_4_(Py)(Pype)]·4Py·11H_2_O (**I**) in a form of polycrystals, suitable for single-crystal X-ray diffraction analysis. A mixture of pyridine–piperidine was used as a solvent, since similar compounds were previously obtained by adding pyridine and triphenylphosphine to a white precipitate, which indicates the need for the presence of strong bases of a donor nature to convert the resulting compound into a soluble form [63]. The reaction proceeds in air without any precautions about daylight. Decanted and twice washed with cold distilled water, the major product was isolated in a 90% yield.

**Figure 1:**
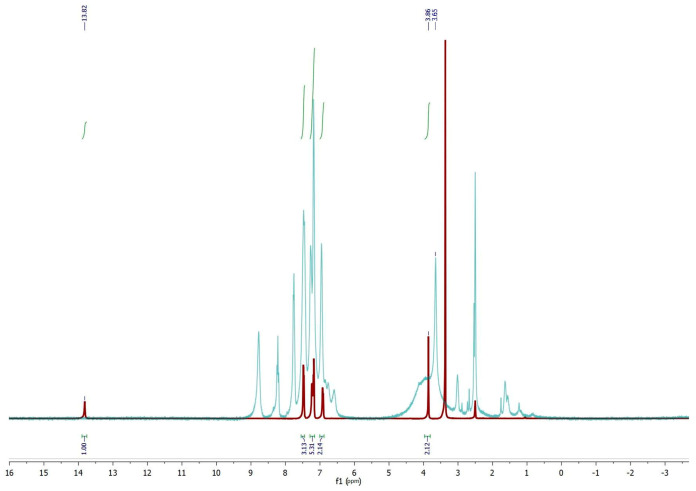
^1^H NMR spectra of **I** (blue line) and free ligand (red line).

The ^1^H NMR spectrum of **I** (Figure 1) is in agreement with the structural data. There is no signal of an SH proton in the ^1^H NMR spectrum of **I**, which confirms the deprotonation of the ligand. The signal of CH_2_ protons is upfield shifted as compared to that one for the free ligand (3.65 and 3.86 ppm, respectively—see also Supplementary Materials). The signals from CH of pyridine (7.6–9.0 ppm) and the CH_2_, NH groups of piperidine (1.2–3.1 ppm) are also observed in the ^1^H NMR spectrum. The ^13^C NMR spectrum of **I** could not be recorded due to the low solubility of the compound in the common deuterated solvents. When trying to accumulate data to record a ^13^C NMR spectrum over a 24 h period, we also discovered that a white precipitate formed in the NMR ampoule. It is likely that deuterated dimethyl sulfoxide molecules replace pyridine and piperidine, reducing the solubility of complex **I**.

### 2.2. Molecular Structure of **I**

**Figure 2:**
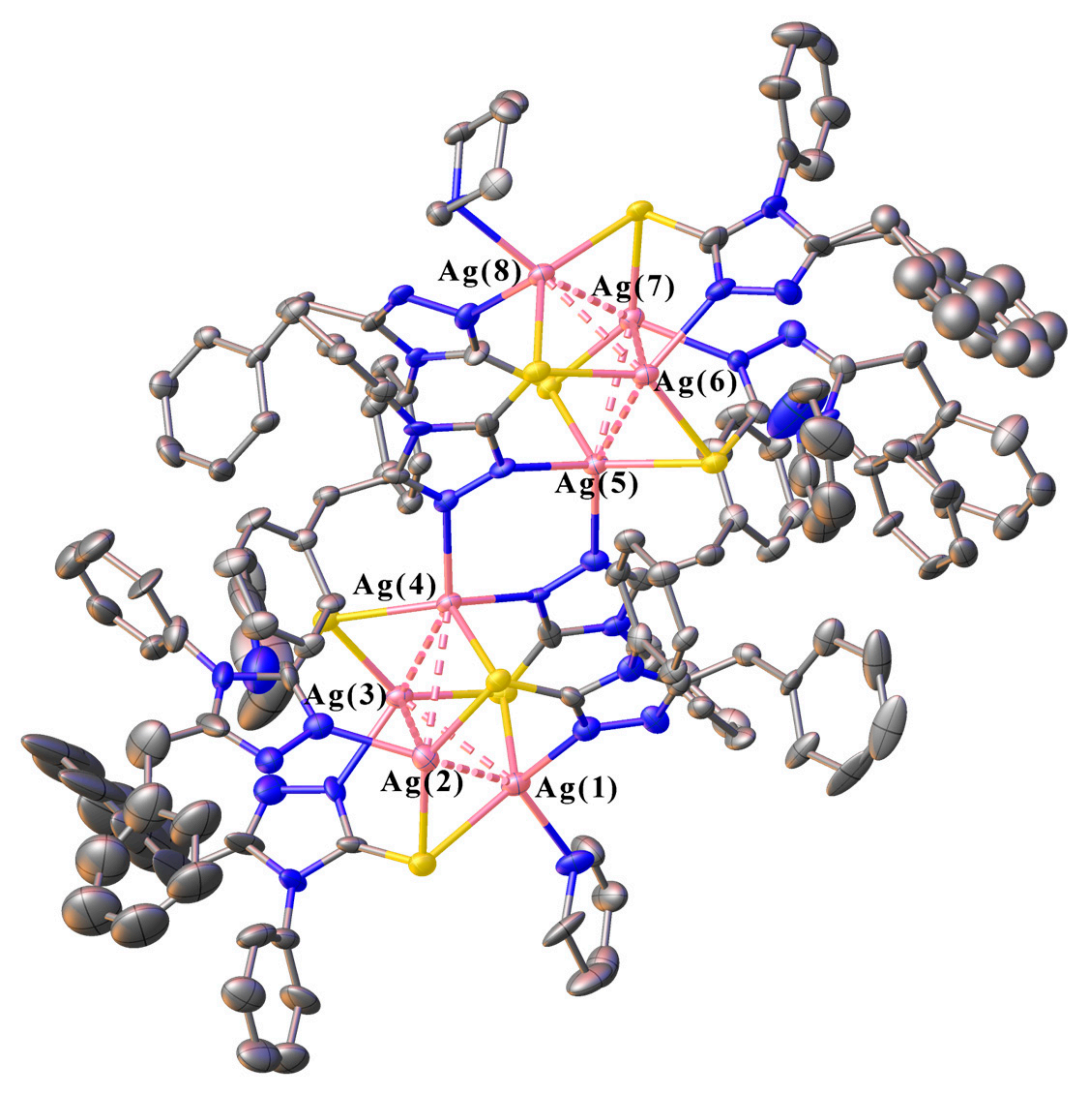
Molecular structure of **I**. Thermal ellipsoids are drawn at 30% probability level. Hydrogen atoms are omitted for clarity. Argentophilic interactions are shown in dotted lines.

**Figure 3:**
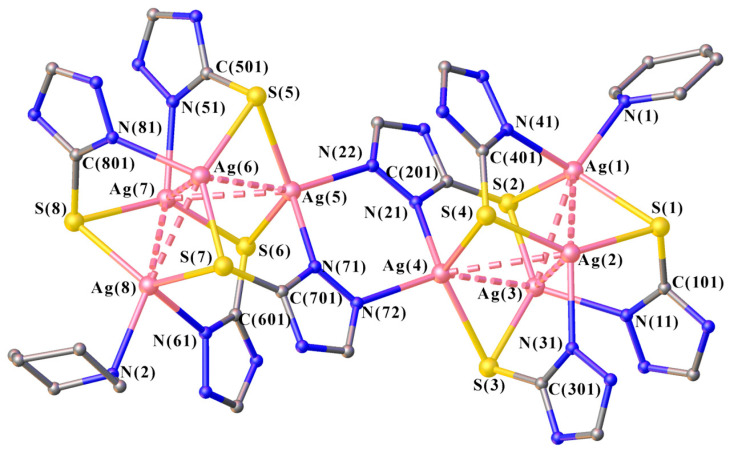
Molecular structure of **I**. Hydrogen atoms, solvate pyridines, and benzyl and phenyl substituents of the ligand have been omitted for clarity. Argentophilic interactions are shown in dotted lines.

Complex **I** crystallizes in the monoclinic space group *Pc* in a form of crystal solvate [Ag_8_L_4_(Py)(Pype)]·4Py·11H_2_O. Since the solvate molecules of pyridine in the crystal are in strong thermal vibrations, only two solvate pyridine molecules were clearly identified, while two other molecules were taken into account as diffuse scattering using the SQUEEZE procedure [64]. Complex **I** is a dimer of two butterfly-type silver-metal clusters (Figure 2 and Figure 3), in which all Ag···Ag distances lie in the range of 2.969(2)–3.254(2) Å. These distances are significantly greater than the sum of the covalent 2.7 Å radii of silver and can apparently be considered as weak secondary interactions (argentophilic) [65,66].

Non-bonding distance between the tips of the “butterfly’s wings” (Ag1···Ag4 4.133(2) Å, Ag5···Ag8 4.236(2) Å) is significantly greater than the distance between atoms at the base of the “butterfly wings” (Ag···Ag 3.137(2), 3.085(2) Å). The outer vertices of the “butterfly wings” (Ag1 and Ag8) coordinate additional monodentate N-donor ligands, pyridine and piperidine, in the presence of which the synthesis and crystallization were carried out (Ag1–N1 2.441(12) Å, Ag8–N2 2.365(15) Å). The inner tips of the “butterfly wings” (Ag4 and Ag5) further coordinate the unsubstituted nitrogen atom in the five-membered triazol ring of one of the ligands in the adjacent butterfly to form a dimeric structure (Ag4–N72 2.389(12) Å, Ag5–N22 2.392(12) Å).

**Figure 4:**
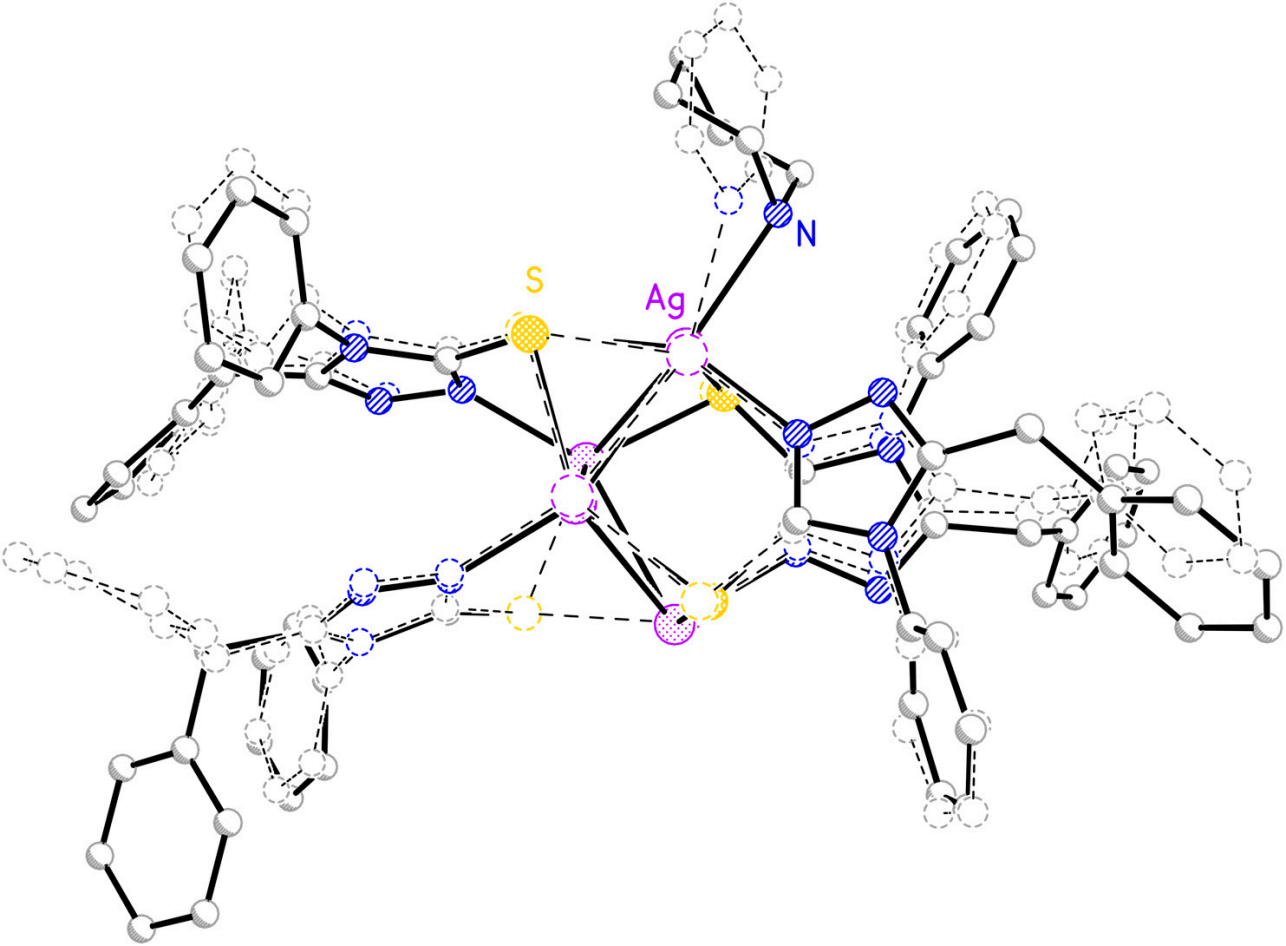
Superposition of a four-nuclear fragment of dimer **I** (solid line) onto another four-nuclear fragment (dashed line) via an inversion pseudocenter; the standard deviation for silver and sulfur atoms is 0.09 Å.

This molecule is pseudocentrosymmetric with the center of inversion at the center of mass of the silver atoms. The structural similarity of the two butterflies that make up the dimer is especially clearly visible when they are superimposed on each other (Figure 4). There is an almost complete coincidence of the positions of all atoms, with the exception of some differences in the orientation of the terminal benzyl substituents, as well as the difference between Py and Pype, which are coordinated by silver atoms.

Four silver atoms of one part of the molecule coordinate four ligands, with each ligand bonded to three metal atoms: the nitrogen atom is bonded to one metal atom (Ag–N 2.275(13)–2.358(15) Å), and a sulfur atom connects two other metal atoms (Ag–S 2.421(4)–2.670(4) Å). The silver atoms at the base of the butterfly have a planar trigonal coordination AgNS_2_ (CN = 3), which is typical for silver clusters formed by a ligand containing an SCN fragment, where the sulfur atom is exocyclic and the CN is endocyclic, while the silver atoms at the tops of the butterfly wings, due to an additional coordination with the N-donor fragments, have a tetrahedral coordination AgN_2_S_2_ (CN = 4). “Butterfly” clusters are not typical for silver with this type of ligands, and in CCDC, there is only one similar structure obtained by Robert P. Lattimer et al. [63]. Much more often a six-nuclei “Cyclohexane-Chair”-type cluster is formed [67,68].

### 2.3. Luminescent Properties

To the best of our knowledge, the luminescent properties of known silver(I) complexes have been studied mainly for square-planar polyhedrals [69], while the studied **I** contains two types of Ag^+^ cations with different environments—triangular and distorted tetrahedral.

The excitation spectrum of solid ligand L at room temperature at λ_em_ = 490 and 510 nm gives a set of bands of 200–430 nm with a maxima at 238, 253, 281, and 400 nm, respectively (Figure S6a). In the emission spectra at λ_ex_ = 240, 250 and 280 nm, a set of bands is observed in the region of 350–650 nm (Figure S6a), where the most intense bands have a maxima at 491, 509, and 530 nm. The excitation spectrum (λ_em_ = 400 nm) of **I** under similar conditions is represented by broad bands in the region of 200–350 nm with a maxima at 240, 255, and 335 nm (Figure S6b). The emission spectrum of **I** at λ_ex_ = 255 nm contains wide bands in the region of 300–600 nm with a maxima at 405, 422, and 487 nm. An analysis of the photophysical properties showed that the transition from ligand L to complex **I** is accompanied by a change in emission as a result of coordination of the ligand to the metal and/or transition to the anionic form. The relative emission intensity of the ligand, as well as its complex, is low.

### 2.4. Biological Activity

The biological activity of **L** and **I** was studied using the nonpathogenic mycobacterial strain *Mycolicibacterium smegmatis*. In mycobacterial strains, the resistance to chemical treatment agents is attributed to the low permeability of its cell wall due to its unusual structure. *M. smegmatis* is a rapidly growing nonpathogenic bacterium, which is therefore used as a model for slowly growing *Mycobacterium tuberculosis* and for the screening of antituberculosis drugs [70]. The *M. smegmatis* test system exhibits higher resistance to antibiotics and antituberculosis agents than *M. tuberculosis*; therefore, the concentration <100 nmol/disc in contrast to *M. tuberculosis* (<4 µg/mL) is used as the selection criterion [71]. All the results obtained for the in vitro biological activity of the compounds under study were compared with the activity of the first-line antituberculosis drug, Rifampicin (**Rif**), under the selected conditions of the experiment. The results of the antimycobacterial activity study are presented in Table 1.

The data shown in Table 1 demonstrate that ligand **L** does not exhibit significant biological activity against the strain of mycobacterium. In turn, the efficiency of complex **I** is increased by almost twenty times in comparison with the free ligand. Similar effect—an increase in biological activity after complex formation is observed quite often [62,72]. The effectiveness of strain suppression—like the reference drug Rif, **I**—has a bactericidal effect; unlike the previously obtained silver complex [62], it exhibits a bacteriostatic effect (most likely associated with the development of resistance genes of mycobacterium).

## 3. Materials and Methods

### 3.1. General Remarks

The synthesized product **I** is stable to oxygen, moisture, and ambient light. Ligand **L** (3-benzyl-4-phenyl-1,2,4-triazol-5-thiol) was synthesized using the procedure similar to that reported earlier [73], of which its ^1^H and ^13^C NMR spectra (see Supplementary Information) are in good agreement with those reported previously. ^1^H NMR (300 MHz, DMSO-*d*_6_) δ: 3.86 (s, 2H, CH_2_), 6.86–6.99 (m, 2H, C_ar_H), 7.14–7.28 (m, 5H, C_ar_H), 7.42–7.55 (m, 3H, C_ar_H), and 13.82 (broad s, 1H, SH). ^13^C NMR (75 MHz, DMSO-*d*_6_) δ: 31.43, 126.86, 128.29, 128.35, 128.61, 129.27, 129.42, 133.55, 134.54, 151.27, and 167.92.

The IR spectrum of **I** was recorded in the range of 400–4000 cm^–1^ using a PerkinElmer (Waltham, MA, USA) Spectrum 65 spectrophotometer equipped with a Quest ATR Accessory (Specac (Orpington, Kent, UK)). The method of attenuated total reflection (ATR) was used. Excitation and emission spectra of the solid samples were recorded at room temperature in the visible range of the spectrum using a PerkinElmer LS-55 spectrometer.

The ^1^H NMR spectra was registered on a Bruker AVANCE (300 MHz) spectrometer with TMS as the internal reference and DMSO-*d*_6_ as the solvent.

To study the biological activity of the free ligand L and its complex **I**, the paper disk method was employed using the nonpathogenic *M. smegmatis mc^2^ 155* strain. A solution of each substance was prepared as follows: a sample of the substance was dissolved in dimethyl sulfoxide to a concentration of 100 mmol or less, depending on the solubility of the substance. Then, tenfold and twofold dilutions of the resulting solution were made and applied to paper disks in an amount of 5 μL/disk. The test method involved measuring the size of the inhibition zone around paper discs containing a compound, which were placed on an agar plate and then seeded with the strain. The concentration of the compound was varied to determine its effect on the growth of the strain. The bacteria washed off Petri dishes with the Trypton-soy agar M-290 medium (Himedia, Mumbai, India) were grown overnight in the Lemco-TW liquid medium (Lab Lemco’ Powder 5 g·L^−1^ (Oxoid, Basingstoke, UK), Peptone special 5 g·L^−1^ (Oxoid), NaCl 5 g·L^−1^, Tween-80) at +37 °C until the average logarithmic growth phase occurred at optical density OD600 = 1.5, which was then mixed with a molten agar medium M-290 in a ratio of 1:9:10 (culture:Lemco-TW:M-290). The mixture was poured into Petri dishes containing pre-solidified agar medium (M-290) at a concentration of 5 mL/dish. After the top layer of agar had solidified, paper discs impregnated with a solution containing the test compound were placed on top of the plate. The culture was incubated for 24 h at +37 °C. The inhibition zone diameters of *M. smegmatis mc*^2^ *155* growth around the paper disks contained the free ligand L and its complex **I** were determined. The Minimum Inhibiting Concentration (MIC) was equal to the concentration of L and **I** that provided the smallest growth inhibition zone.

X-ray diffraction data for **I** were collected at 150 K with a Bruker Quest D8 CMOS diffractometer, using graphite monochromated Mo-Kα radiation (*λ* = 0.71073 Å, ω-scans). The crystal structure of **I** was solved using Intrinsic Phasing with the ShelXT [74] in Olex2 [75] and then refined with the ShelXL [76] refinement package using Least-Squares minimization against F^2^ in the anisotropic approximation for non-hydrogen atoms. Hydrogen atoms were calculated and they all were refined in the isotropic approximation within the riding model. The contribution of highly disordered solvent molecules in structural voids was treated as diffuse using the SQUEEZE procedure implemented in the PLATON software, version 2023.1 [64]. The electron count within cavities (V1 = 726 Å3 and V2 = 688 Å3) measured 170 and 223 e^−^, respectively; per formula unit of I, their sum corresponds to 11 water and 4 pyridine molecules. The crystal data and structure refinement parameters are given in Table 2. CCDC 2300382 contains the supplementary crystallographic data for this manuscript.

### 3.2. Synthesis of **I**

A portion of AgNO_3_ (0.170 g, 1 mmol) was dissolved in 5 mL of distilled water and then the solution of ligand L (0.270 g, 1 mmol) dissolved in 20 mL of MeOH was added, of which the resulting reaction mixture was heated at 50 °C with constant stirring and the solution gradually began to become white. After half an hour, the reaction mixture was filtered and the resulting white precipitate was dried and then redissolved in 20 mL of a 1:1 pyridine–piperidine mixture; within a week, crystals amenable for single-crystal X-ray diffraction were obtained via slow evaporation in a glass beaker capped with dotted Parafilm. The yield of the reaction is 90%. ATR-IR, ν/cm^–1^: 3434 w, 3057 w, 3032 w, 2919 w, 2847 w, 1631 w, 1593 w, 1524 m, 1496 m, 1424 m, 1367 s, 1311 s, 1245 s, 1160 m, 1069 m, 1031 m, 1009 m, 921 w, 824 w, 758 s, 729 s, 689 vs, 604 m, 557 s, 500 w, 465 w, 418 w, 403 w. ^1^H NMR (300 MHz, DMSO-d_6_) δ: 3.65 (broad s, 16H, CH_2_), 6.47–7.64 (m, 80H, C_ar_H), 7.66–7.90 (m, 8H, C_py_H), 8.14–8.34 (m, 4H, C_py_H), 8.63–8.99 (m, 8H, C_py_H).

## 4. Conclusions

Here, we have presented a method for preparing a new silver(I) complex with 3-benzyl-4-phenyl-1,2,4-triazol-5-thiol and determined its crystal structure. Photophysical studies of **L** and its complex **I** showed weak emission of the organic ligand in neutral and anionic form; the complexation is accompanied by the changes in the emission spectra. The study of the biological activity of **L** and **I** have determined that the free ligand does not exhibit significant bactericidal action against *Mycobacteria smegmatis*, whilst complexation with silver has increased the biological activity of the ligand by 20 times. We believe that our results may encourage chemists to synthesize novel bioactive silver complexes of 1,2,4-triazole derivatives via a facile method in water–methanol mixtures to prepare advanced pharmaceutical products.

## Figures and Tables

**Table 1:** The results of antibacterial activity study of free ligand **L** and complex **I** against *M. smegmatis*.

Compound	MIC, (nmol/disc)	Zone of Inhibition, mm	
	24 h	24 h	120 h
**L**	>1000	0	0
**I**	50	6.5 ± 0.1	6.4 ± 0 *
[Ag_3_(fur)(bpy)_3_]_n_ [62]	15	6.4 ± 0.12	6.2 ± 0 **
**Rif**	5	7.1 ± 0.76	7.0 ± 0.4 *

The diameter of the paper disk is 6 mm. * The *M. smegmatis* growth inhibition zone is transparent, there is no weak background growth of the culture (bactericidal effect). ** *M. smegmatis* growth inhibition zone overgrows (bacteriostatic effect). 0—no growth inhibition zone.

**Table 2:** Crystal data and structure refinement for **I**.

Parameter	Value
Empirical formula	C_150_H_154_N_30_O_11_S_8_Ag_8_
Formula weight	3316.2
Crystal system	Monoclinic
Space group	*Pc*
Z	2
T, K	150
*a* (Å)	23.729(3)
*b* (Å)	25.401(3)
*c* (Å)	12.617(1)
*α* (°)	90
*β* (°)	93.340(4)
*γ* (°)	90
V (Å^3^)	7591.9(14)
*D*_calc_ (g/cm^3^)	1.451
*μ*(Mo-K_α_) (mm^−1^)	1.17
*F*(000)	3324
2*θ*_max_ (deg.)	50
*R*_int_	0.0988
Reflections measured	137,347
Independent reflections	118,888
Observed reflections [*I* > 2σ(*I*)]	18,266
Parameters/restraints	1447/305
*R*_1_	0.0561
*wR*_2_	0.1470
*GooF*	1.002
*Flack*	0.18(4)
Δ*ρ*_max_/Δ*ρ*_min_ (e/Å^−3^)	1.34/−0.96

## Data Availability

The structure parameters of the obtained compound were deposited with the Cambridge Structural Database (CCDC No. 2300382 (**I**) deposit@ccdc.cam.ac.uk or http://www.ccdc.cam.ac.uk/data_request/cif).

## Supplementary Materials

The following supporting information can be downloaded at https://www.mdpi.com/article/10.3390/molecules29010105/s1: Figure S1: IR-spectrum of **L**; Figure S2: IR-spectrum of compound **I**; Figure S3: IR-spectrum of **L** and compound **I**; Figure S4: ^1^H NMR spectrum of **L**; Figure S5: ^13^C NMR spectrum of **L**; Figure S6: Excitation and emission spectra for solid samples **L** (a) and **I** (b) at room temperature.
