# Supplementary material for: Synthesis, Structure, Biological Activity, and Luminescence Properties of a “Butterfly”-Type Silver Cluster with 3-Benzyl-4-phenyl-1,2,4-triazol-5-thiol
Source: Molecules. 2023 Dec 23;29(1):105. doi: 10.3390/molecules29010105 (PMC10779673; doi:10.3390/molecules29010105)
Supplement: Supplementary file 1 [file molecules-29-00105-s001.zip › molecules-2786172-supplementary.pdf]

molecules MDPI

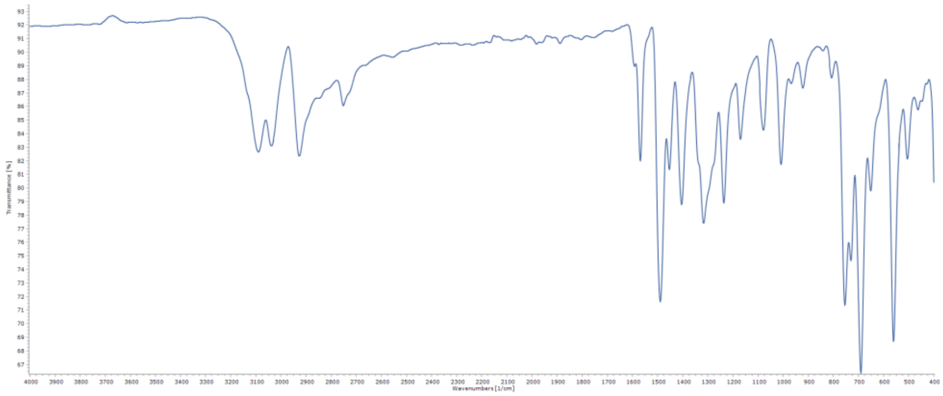

Figure S1. IR-spectrum of 3-benzyl-4-phenyl-1,2,4-triazol-5-thiol (L)

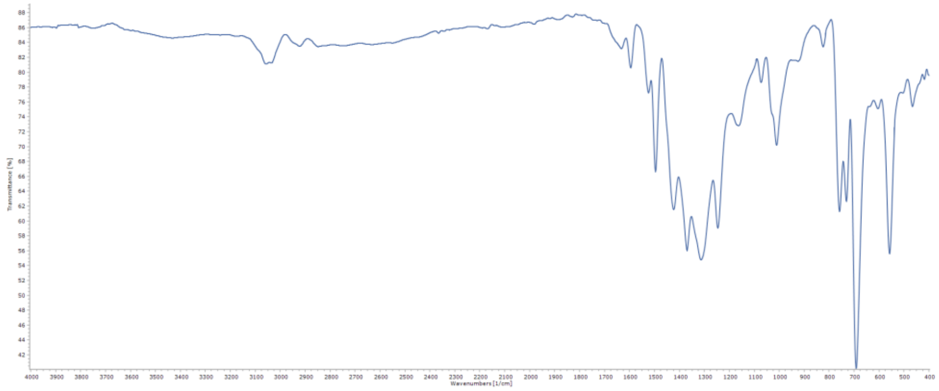

Figure S2. IR-spectrum of **I**

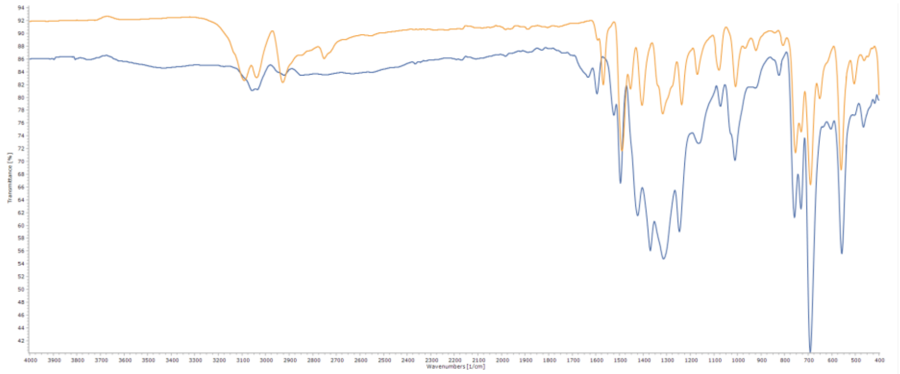

Figure S3. Superposition of the IR-spectra of **I** and L

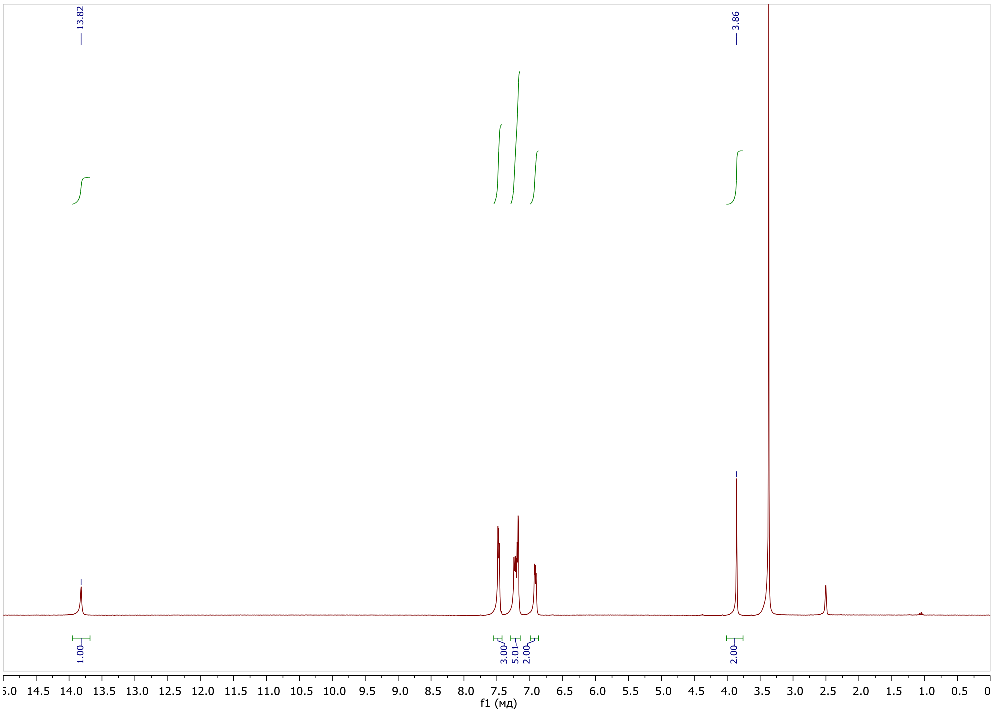

Figure S4. ^{1}H NMR spectra of L

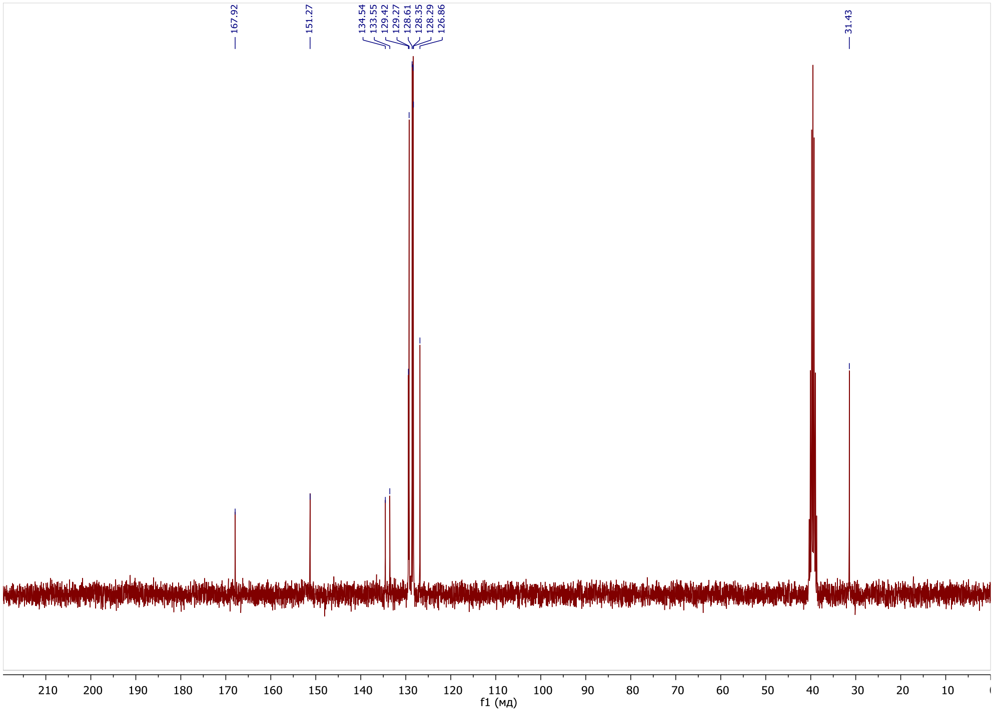

Figure S5. ^{13}C NMR Spectrum of L

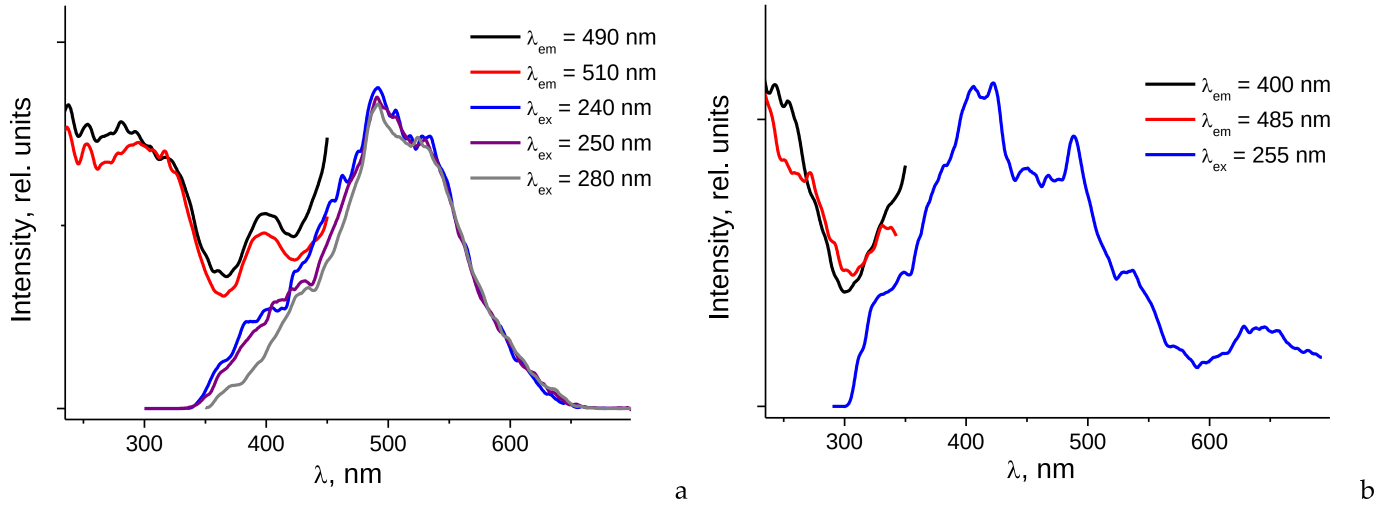

Figure S6. Excitation and emission spectra for solid samples L (a) and **I** (b) at room temperature.
